# Neurovictimology and the risks of neurotechnologies and artificial intelligence: a forensic and legal perspective

**DOI:** 10.3389/fpsyg.2026.1823924

**Published:** 2026-04-16

**Authors:** Eric García-López, Ana Ruth Díaz-Victoria, Lorena Contreras-Taibo, Cristina Nombela

**Affiliations:** 1 Instituto Nacional de Ciencias Penales, Mexico City, Mexico; 2 Unidad de Cognición y Conducta, Instituto Nacional de Neurología y Neurocirugía Manuel Velasco Suárez, Mexico City, Mexico; 3 Universidad Diego Portales, Santiago de Chile, Chile; 4 Biological and Health Psychology Department, Universidad Autónoma de Madrid, Madrid, Spain

**Keywords:** cognitive liberty, forensic psychology, neuroethics, neurolaw, neurorights, neurotechnology, neurovictimology

## 1 Introduction

The encounter between law and neuroscience has opened a profoundly reconfigured intellectual terrain —one in which the once-stable borders separating biological processes, moral agency, and juridical responsibility appear increasingly permeable (Greene and Cohen, 2004; Morse, 2006). What was long treated as a philosophical question about free will or intent now unfolds against the backdrop of brain scans, neural modulation techniques, and computational models capable of interpreting cognitive patterns (Schleim, 2024). In this context, particularly as fMRI moves into real-world settings, there is a risk that neuroimages are treated as more conclusive than they are—taken as direct and definitive windows into the mind rather than as complex, interpretive data that require careful contextualization. As Schleim and Roiser (2009) already cautioned nearly two decades ago, such images can easily acquire a misplaced aura of objectivity that obscures the interpretive layers behind their production.

Over the past two decades, the accelerated development of neurotechnologies—ranging from non-invasive brain stimulation and advanced neuroimaging to artificial intelligence–mediated brain–computer interfaces—has compelled legal systems to revisit some of their most foundational assumptions (Ienca and Andorno, 2017). Concepts such as autonomy, mental privacy, and culpability can no longer be understood solely in normative abstraction (Farahany, 2012); they must now be examined in light of technologies that can observe, influence, and potentially reshape the neural substrates of decision-making itself (UNESCO, 2022, 2023; Yuste et al., 2017).

Artificial intelligence intensifies this transformation. As machine-learning systems are increasingly used to classify neural signals, predict behavioral tendencies, or assist in forensic evaluation (Tortora et al., 2020), the interpretation of brain data is no longer exclusively a clinical or judicial act but an algorithmic one. Artificial intelligence does more than process data; it selects patterns, assigns probabilistic weight, and frames what counts as relevant. In forensic neuroscience, this framing power can influence how neural evidence is interpreted before it reaches judicial reasoning. As research in deep learning has shown, these systems generate predictive architectures that shape how data are classified and how inferences are drawn (LeCun et al., 2015).

## 2 Defining neurovictimology

Although neurolaw and neuroethics have carefully examined the implications of neuroscience for responsibility and rights (Farah, 2012), they have largely overlooked a decisive figure in this transformation: the victim. Neurovictimology emerges precisely to address this lacuna. The term was introduced in Spanish by García-López et al. (2023) in a conceptual formulation, where it was proposed as an integrative framework uniting victimology, neuroscience, and forensic-legal analysis, from which neurovictimology emerges as the systematic study of the neural sequelae of victimization, encompassing structural and functional brain alterations and their emotional, cognitive, and behavioral correlates within defined environmental contexts. As an interdisciplinary field integrating empirical neuroscience and normative legal analysis, it examines how trauma, neural vulnerability, and neurotechnological exposure configure victimhood in both biological and juridical registers. Grounded in classical victimology, it extends inquiry beyond social and psychological determinants of harm to the neural substrates of memory, affect regulation, resilience, and testimonial competence.

At its core, neurovictimology integrates three dimensions. Scientifically, it employs neuroimaging, psychophysiological assessment, and neurocognitive models to understand the cerebral impact of violence and coercion (Hinojosa et al., 2024). Legally, it scrutinizes the use—and potential misuse—of brain-based evidence within judicial proceedings (Aono et al., 2019; Nita, 2015). Ethically, it examines whether these practices truly protect cognitive liberty, mental privacy, and personal identity (Ienca and Andorno, 2017). Such risks may be particularly acute in regions marked by institutional fragility and pronounced social asymmetry, including parts of Latin America, where regulatory frameworks are still evolving (Andorno, 2023) and neurolaw and neurorights are still in an early but steadily consolidating phase of development (García-López et al., 2019).

Neurovictimology must extend beyond mapping the neural consequences of trauma and confront a more exacting question: under what conditions does neuroscientific practice itself risk generating harm? The concern is not only how violence alters neural architecture, but what ensues when patterns of brain activity are elevated to evidentiary authority (Farah, 2014). As Phelps and Hofmann (2019) review, mechanisms of memory consolidation and reconsolidation—once largely confined to speculative fiction—are now the subject of experimental and early translational research aimed at attenuating or modifying emotional and traumatic memories. Although clinical efficacy remains variable and context-sensitive, the rapid development of these interventions marks a transition from imaginative projection to controlled neurobiological manipulation, carrying substantial epistemic and normative consequences.

When credibility is inferred from imaging, when neural data are collected within asymmetrical power structures, or when neuromodulation is deployed in coercive contexts, the individual risks epistemic displacement, becoming less a speaking subject and more an object of measurement (Ligthart, 2019). The integration of artificial intelligence sharpens this shift: algorithmic systems classify and predict from neural data, subtly redistributing interpretive power and complicating responsibility (Ienca et al., 2022). What appears as epistemic refinement can entail a contraction of personhood. This is not a peripheral technical matter but a juridical threshold. Technologies that promise objectivity may also displace context and meaning (Illes and Racine, 2005).

In this regard, neurovictimology has begun to interrogate how neurotechnological tools themselves can operate as vectors of harm: for instance, the use of functional neuroimaging techniques such as functional MRI to assess a victim's credibility in court risks reducing complex affective and experiential states to discrete neural correlates, thereby distorting the phenomenological depth of suffering, echoing concerns raised by Greely (2011) regarding the epistemic and normative limits of forensic neuroimaging.

In unequal environments, their use may do more than expose vulnerability—it may consolidate it under the mantle of scientific neutrality. The rapid advance of brain–computer interfaces, sophisticated imaging, neuromodulatory techniques, and AI-driven analytics has expanded both therapeutic promise and forensic ambition. Artificial intelligence systems increasingly mediate the interpretation of neural data, generating probabilistic inferences about risk, credibility, or intent. Yet algorithmic opacity, biased training data, and automation bias can reproduce and amplify distortions affecting historically excluded populations, while diffusing accountability across technical infrastructures (McNellan et al., 2022). At the same time, these tools complicate the boundary between voluntary agency and technologically influenced mental states, particularly in criminal justice and other asymmetrical settings. This shift is not merely theoretical; it can intensify existing vulnerabilities and generate forms of injustice that are technocratic, distributed, and difficult to contest precisely because they appear computationally objective (Benjamin, 2019). The concern is reinforced by evidence that brain development is shaped by parentification and chronic adversity (Schleim, 2025) affecting stress regulation, executive functioning, and emotional processing. When such developmentally patterned features are treated as isolated neural indicators within predictive systems, structural disadvantage risks being recoded as individual pathology.

Thus understood, neurovictimology rests on two fundamental propositions: first, that brain-based evidence can illuminate the neurobiological impact of harm; and second, that neurotechnologies themselves can generate new forms of victimhood through intrusion into mental autonomy and integrity. By foregrounding these dynamics, neurovictimology invites the legal system to recognize that harm may occur not only through physical violence or social coercion, but through interventions—direct or mediated—within the neural substrate of the person. A neurovictimological framework also introduces a dimension that may complicate legal analysis: the neurofunctional substrates of resilience (Moreno-López et al., 2020). Some victims do not exhibit clinically detectable psychological sequelae but instead develop adaptive coping mechanisms that preserve functional capacity (van Rooij et al., 2024).

This distinction may be understood in terms of differential neurobiological patterns, including predominant limbic dysregulation—commonly associated with depression, anxiety, and post-traumatic stress disorder—vs. stronger prefrontal cortical regulation, which underlies executive control, emotional modulation, and resilient adaptation.

## 3 Neurotechnological risks and ethical challenges

Neurotechnologies, once largely confined to research settings, now extend across commercial, military, and clinical domains. Their diffusion expands therapeutic possibilities while increasing ethical exposure. The most acute risks implicate neurorights—mental privacy, cognitive freedom, and psychological continuity (Ligthart et al., 2023; García-López et al., 2021). While many of these harms remain anticipatory, from a neurovictimological perspective they delineate plausible loci of harm, where technological intervention may itself operate as a vector of victimization.

Analogous developments in adjacent technological domains render these risks more concrete. Documented forms of algorithmic discrimination in decision-making systems (Wang et al., 2024) and measurable performance disparities in AI-assisted cancer diagnosis across demographic groups (Lin et al., 2025) demonstrate how data-driven technologies can reproduce and operationalize structural inequities. Likewise, the forensic use of pacemaker data to challenge a defendant's account (Maras and Wandt, 2020) raises salient concerns regarding privacy and the privilege against self-incrimination in relation to data generated by one's own body. While not strictly neurotechnological, such precedents substantiate the plausibility and near-term relevance of comparable harms in the neurotechnology domain.

### 3.1 Cognitive privacy and data exploitation

Neurodevices that decode or modulate mental states enable surveillance at the neural level. Unlike conventional data, neural data implicates memory, intention, and identity. As noted by Farina and Lavazza (2022), such technologies may expose or alter memory traces, raising risks of manipulation and coerced self-incrimination. Unauthorized neural access thus constitutes a direct form of psychological harm.

### 3.2 Informed consent and autonomy

Neurotechnological interventions may influence cognition outside conscious awareness. Adenzato et al. (2019) showed that transcranial direct-current stimulation can affect social cognition and Theory of Mind, potentially compromising decisional autonomy and the validity of consent in coercive or weakly regulated settings.

### 3.3 Neurodiscrimination and inequality

Enhancement technologies risk amplifying structural inequalities (Lavazza, 2018). Differential access may entrench disadvantage, while neurocognitive metrics can enable discriminatory practices, including “neuroprofiling” (Webb et al., 2022).

### 3.4 Weaponization

Military applications of neurotechnology, including brain–computer interfaces and cognitive modulation, raise concerns about coercive manipulation and human rights violations (Giordano, 2012; Ienca and Andorno, 2017).

Across these domains, neurovictimology functions as an analytical framework to identify, classify, and prevent neural forms of harm, integrating forensic accountability with neuroethical regulation.

## 4 Forensic and legal implications

Neuroscientific evidence has reshaped doctrines of responsibility and culpability while exposing limits in victim protection. Neurovictimology refocuses attention on victims' evidentiary position and mental integrity, yet existing legal frameworks offer only partial safeguards. Neuroimaging and neuropsychological assessment may corroborate trauma [e.g., PTSD (Post-traumatic stress disorder), TBI (Traumatic brain injury)], but also risk overinterpretation, reductionism, and intrusion into mental privacy, requiring methodological rigor and strict ethical limits (Ligthart et al., 2023; Ligthart, 2019). The recognition of neurorights, reflected in Chile's 2021 constitutional reform and legislative initiatives in Mexico (Herrera-Ferrá et al., 2025), signals a structural shift, framing cognitive liberty, mental privacy, and identity as protected interests and requiring safeguards against misuse of neurodata and intrusive interventions (Aggarwal and Jain, 2024; De Marco, 2022). Beyond the brain, neurovictimology must also address harms involving peripheral, enteric, and neuroendocrine systems, as victimization (especially when chronic) produces distributed biological effects (Mayer et al., 2015; McEwen, 2007).

## 5 Latin American context

Latin America may assume a central role in neurolaw and neurorights, grounding neuroethics in human dignity and social justice amid structural inequality (García-López and Muñoz, 2025; Herrera-Ferrá et al., 2025). In this context, the expansion of neurotechnologies (often via public or state-led initiatives) raises acute concerns about oversight and the protection of vulnerable groups. Chile's constitutional recognition of neurorights and emerging efforts in Mexico illustrate normative leadership, but convergence with AI complicates the landscape: systems interpreting neural data may embed bias, diffuse responsibility, and channel brain data into automated decisions. The challenge is thus not only to proclaim neurorights, but operationalize them through enforceable standards and algorithmic transparency, lest protections remain aspirational and remedies limited.

## 6 Discussion and future directions

Neurovictimology is not ancillary to victimology or neuroethics; it extends both by treating the brain as a locus and target of harm, compelling law and forensic practice to reassess injury, evidence, and protection in technologically mediated contexts (Greely, 2011).

Its next phase must be operational. Clear conceptual and diagnostic criteria are needed to identify neurotechnological victimization, integrating neurobiological findings with disciplined psychological and contextual analysis. Standardized protocols should govern the collection, storage, and interpretation of neural data, aligned with emerging neurorights frameworks. Legal and mental-health professionals must be trained in the evidentiary limits and ethical risks of brain-based evidence, and regulatory coordination is necessary to avoid fragmentation.

AI systems processing neural data must be auditable, bias-controlled, and tied to clear lines of responsibility; otherwise, algorithmic mediation risks obscuring error and diffusing accountability.

At its core, neurovictimology is not about multiplying categories of victims. It is about recalibrating justice in a world where mental processes can be recorded, modeled, and potentially altered. If mental privacy and cognitive liberty are to stand alongside bodily integrity as foundational legal goods (Ienca and Andorno, 2017), then the protection of persons must extend to the neural substrate of agency itself.

The true horizon of human rights is no longer only external conduct or physical restraint. It now reaches inward. Neurovictimology names that frontier—and insists that law arrives there before harm does.
